# Supplementary figures and images for: Single-molecule lipid biosensors mitigate inhibition of endogenous effector proteins
Source: J Cell Biol. 2025 Feb 11;224(3):e202412026. doi: 10.1083/jcb.202412026 (PMC11812570; doi:10.1083/jcb.202412026)

Fig. 1B

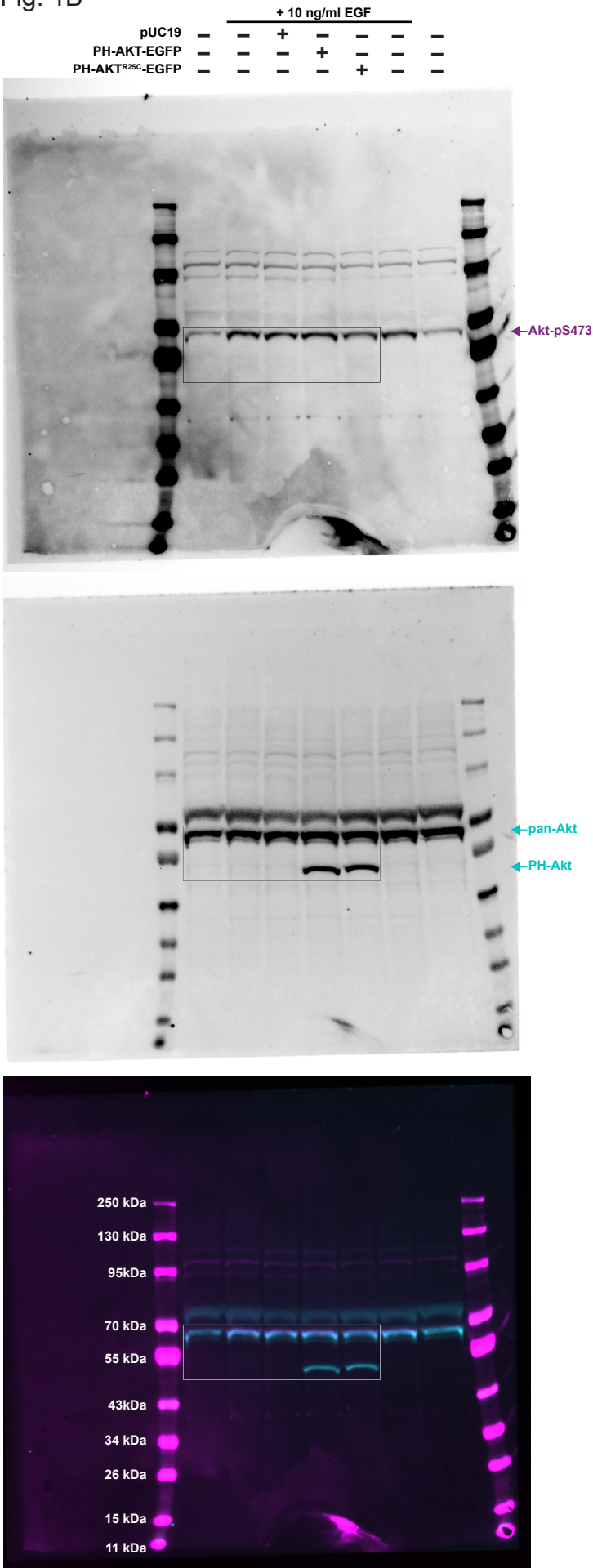

Supplement: SourceData F1 — is the source file for Fig. 1. [file jcb_202412026_sourcedataf1.pdf]

Source Data F2

Fig. 2B

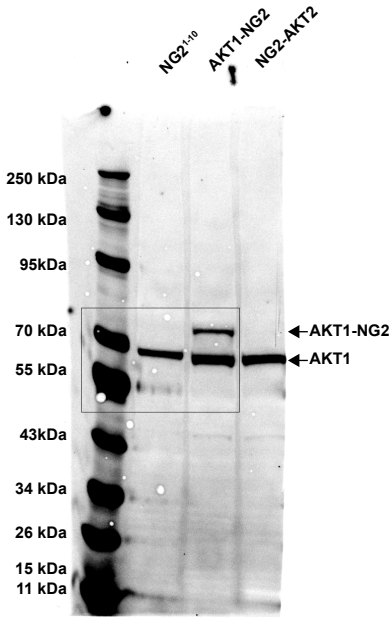

Supplement: SourceData F2 — is the source file for Fig. 2. [file jcb_202412026_sourcedataf2.pdf]
